# (4-Carbamoylphen­yl)boronic acid

**DOI:** 10.1107/S1600536810014789

**Published:** 2010-05-08

**Authors:** Margarita D. Apostolova, Rositsa P. Nikolova, Boris L. Shivachev

**Affiliations:** a Institute of Molecular Biology "Acad. R. Tsanev", Bulgarian Academy of Sciences, Acad G. Bonchev Str. Building 21, 1113 Sofia, Bulgaria; b Central Laboratory of Mineralogy and Crystallography, Bulgarian Academy of Sciences, Acad G. Bonchev Str. Building 107, 1113 Sofia, Bulgaria

## Abstract

In the title compound, C_7_H_8_BNO_3_, the mol­ecule lies on an inversion center leading to a statistical disorder of the B(OH)_2_ and CONH_2_ groups. In the crystal structure, mol­ecules are linked by N—H⋯O and O—H⋯O hydrogen bonds, forming sheets parallel to the *bc* plane. The B(OH)_2_ and CONH_2_ groups are twisted out of the mean plane of the benzene ring by 23.9 (5) and 24.6 (6)°, respectively.

## Related literature

For general background to the use of boronic acids in organic synthesis, as pharmaceutical agents and in crystal engineering see: Miyaura & Suzuki (1995 ▶); Suzuki (1999 ▶); Adams & Kauffman (2004 ▶); Barth *et al.* (2005 ▶); Minkkilä *et al.* (2008 ▶); Maly *et al.* (2006 ▶); Desiraju (1995 ▶); James *et al.* (2006 ▶).. For related structures, see: Cobbledick & Small (1972 ▶); Rodríguez-Cuamatzi *et al.* (2004 ▶). For hydrogen-bond motifs, see: Bernstein *et al.* (1995 ▶).
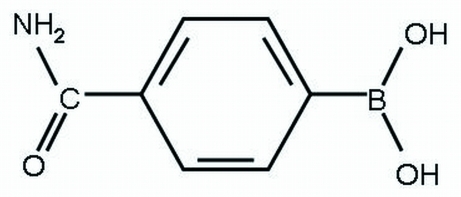


## Experimental

### 

#### Crystal data

C_7_H_8_BNO_3_
*M* *_r_* = 164.95
Triclinic, 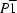

*a* = 4.997 (2) Å
*b* = 5.351 (2) Å
*c* = 7.2967 (16) Å
α = 103.912 (13)°
β = 98.69 (2)°
γ = 93.136 (14)°
*V* = 186.36 (11) Å^3^
*Z* = 1
Mo *K*α radiation
μ = 0.11 mm^−1^
*T* = 290 K
0.27 × 0.25 × 0.25 mm

#### Data collection

Enraf–Nonius CAD-4 diffractometer
2155 measured reflections
1078 independent reflections
755 reflections with *I* > 2σ(*I*)
*R*_int_ = 0.054
3 standard reflections every 120 min  intensity decay: 2%

#### Refinement

*R*[*F* ^2^ > 2σ(*F* ^2^)] = 0.053
*wR*(*F* ^2^) = 0.148
*S* = 1.03
1078 reflections
75 parameters
88 restraints
H-atom parameters constrained
Δρ_max_ = 0.28 e Å^−3^
Δρ_min_ = −0.23 e Å^−3^

### 

Data collection: *CAD-4 EXPRESS* (Enraf–Nonius, 1994 ▶); cell refinement: *CAD-4 EXPRESS*; data reduction: *XCAD4* (Harms & Wocadlo, 1995 ▶); program(s) used to solve structure: *SHELXS97* (Sheldrick, 2008 ▶); program(s) used to refine structure: *SHELXL97* (Sheldrick, 2008 ▶); molecular graphics: *ORTEP-3 for Windows* (Farrugia, 1997 ▶) and *Mercury* (Bruno *et al.*, 2002 ▶); software used to prepare material for publication: *WinGX* (Farrugia, 1999 ▶).

## Supplementary Material

Crystal structure: contains datablocks I, global. DOI: 10.1107/S1600536810014789/lh2998sup1.cif

Structure factors: contains datablocks I. DOI: 10.1107/S1600536810014789/lh2998Isup2.hkl

Additional supplementary materials:  crystallographic information; 3D view; checkCIF report

## Figures and Tables

**Table 1:** Hydrogen-bond geometry (Å, °)

*D*—H⋯*A*	*D*—H	H⋯*A*	*D*⋯*A*	*D*—H⋯*A*
O1—H1⋯O2^i^	0.82	1.96	2.77 (2)	167
O2—H2*A*⋯O1^ii^	0.82	2.05	2.79 (2)	149
O2—H2*A*⋯O3^iii^	0.82	2.00	2.73 (2)	149
N1—H1*A*⋯O3^iv^	0.86	2.14	2.97 (3)	160.7
N1—H1*B*⋯O1^v^	0.86	2.30	2.97 (2)	135.7
N1—H1*B*⋯O3^vi^	0.86	2.18	2.90 (2)	140.8

Symmetry codes: (i) 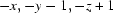; (ii) 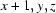; (iii) 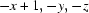; (iv) 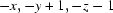; (v) 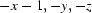; (vi) 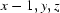.

**Fig. 1.**
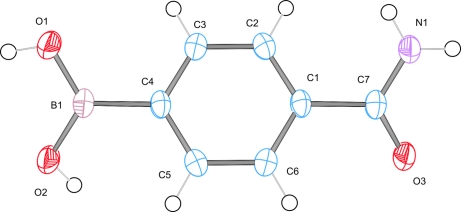
The molecule of title compound with the atom numbering scheme showing 50% probability displacement ellipsoids. H atoms are shown as small spheres of arbitrary radii.

**Fig. 2.**
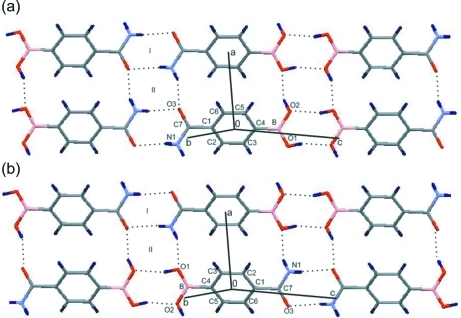
Two possible ways of molecular arrangement in the unit cell, showing the hydrogen-bonding interactions as dashed lines: type I connects opposite sides of molecules to chains and II links the chains together.

**Fig. 3.**
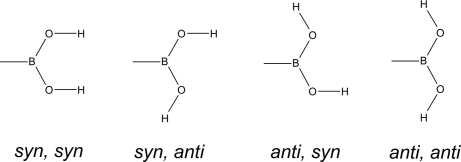
Possible conformations of the B(OH)2 functional group.

**Fig. 4.**
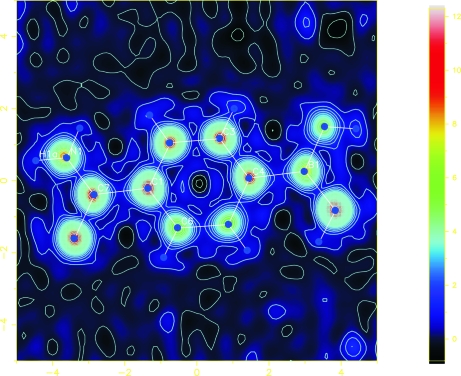
Fo electron density viewed perpendicular to the mean plane of the molecule.

## supplementary crystallographic information

### Comment

The title compound possesses two distinct functional groups: boronic acid and amide. Compounds containing the boronic acid moiety are important as precursors for organic transformations (Miyaura & Suzuki, 1995; Suzuki, 1999;) and recently attention has been focused on these types of compounds as potential pharmaceutical agents (Adams & Kauffman, 2004; Barth *et al.*, 2005; Minkkilä *et al.*, 2008). Amides are versatile precursors to many other functional groups and undergo many chemical reactions, usually through an attack on the carbonyl group. The title compound is a commercial product and we solved its crystal structure to verify the repeatability of the weak interactions already observed in the structures of terephthalamide and phenylboronic acid Cobbledick & Small, 1972; Rodríguez-Cuamatzi, P. *et al.*, 2004. Self assembling based on hydrogen-bonding motifs is of general interest for crystal engineering, structural chemistry and biology (Maly *et al.*, 2006; Desiraju, 1995).

The crystal structure of the studied compound contains molecules linked together by hydrogen bonds in sheets similar to those of terephthalamide (Cobbledick & Small, 1972) and 1,4-phenilboronic acid (Rodríguez-Cuamatzi *et al.*, 2004) (Fig. 1). More over all tree compounds have similar triclinic lattice parameters and crystallize in the centrosymmetric P-1 space group. In the title compound, the location of the molecule on a center of symmetry leads to a statistical disorder of the B(OH)_2_ and CONH_2_ groups (Fig. 1). The B(OH)_2_ and CONH_2_ groups are out of the mean plane of the benzene ring by 23.9 (5)° and 24.6 (6)° respectively. Similar angle is reported for the amide group in terephthalamide (23°) while the one for 1,4 phenilboronic acid is greater (~35°). It should be noted that C—C (phenyl-amide) and C—B distances of 1.505 (6) Å and 1.546 (6)Å are restrained to match those in the terephthalamide molecule C—C (phenyl-amide) distance of 1.489 (5) Å and that of the 1,4-phenilboronic acid molecule with C—B of 1.564 (3) Å.

Both amide and boronic acid groups are involved in hydrogen bonds to form ring motifs marked by I and II (Fig. 2). Type I, R^2^_2_(8) (Bernstein *et al.* 1995) connects opposite sides of molecules to chains. Type II links the chains to form sheets parallel to *bc*. However, two type of motifs linking the chains can be proposed: R^4^_4_(8) (Fig. 2a) and R^3^_4_(8) (Fig. 2b). Indeed, hydrogen bonding pattern can vary depending on the position of the hydrogen atoms attached to the B(OH)_2_ moiety (Fig. 3). The current position of H atoms for the B(OH)_2_ group (*syn, anti*) results from a SHELX AFIX 147 instruction. As a result the bonding interaction between the B(OH)_2_ and amide groups is forbidden, due to the short contact between hydrogen atoms linked to O1 and N1 (H1···H1A 1.272 Å). Thus the hydrogen bonding interactions in the chains are limited to "boronic-boronic" and "amid-amide". An alternative (*anti, syn*) positioning for H attached to O will permit hydrogen bonding between B(OH)_2_ and amid groups but an F_o_ map (Fig. 4) does not suggest an (*anti, syn*) conformation for the H atoms.

### Experimental

The studied compound is a commercial product (Frontier Scientific). Colorless crystals of C_7_H_8_NBO_3_, were obtained after several days staying from 50% water:ethanol solution at 277K.

### Refinement

All H atoms were placed in idealized positions (C—H = 0.93 Å, O—H = 0.82 Å and N—H = 0.86 Å) and were constrained to ride on their parent atoms, with U_iso_(H) = 1.2U_eq_(C, O or N). Disorder refinement required the introduction of appropriate series of restraints on bond lengths and planarity.

### Crystal data

**Table:**

C_7_H_8_BNO_3_	*Z* = 1
*M**_r_* = 164.95	*F*(000) = 86
Triclinic, *P*1	*D*_x_ = 1.470 Mg m^−^^3^
Hall symbol: -P 1	Melting point: not measured K
*a* = 4.997 (2) Å	Mo *K*α radiation, λ = 0.71073 Å
*b* = 5.351 (2) Å	Cell parameters from 22 reflections
*c* = 7.2967 (16) Å	θ = 18.0–19.8°
α = 103.912 (13)°	µ = 0.11 mm^−^^1^
β = 98.69 (2)°	*T* = 290 K
γ = 93.136 (14)°	Prismatic, colorless
*V* = 186.36 (11) Å^3^	0.27 × 0.25 × 0.25 mm

### Data collection

**Table:**

Enraf–Nonius CAD-4 diffractometer	*R*_int_ = 0.054
Radiation source: fine-focus sealed tube	θ_max_ = 30.0°, θ_min_ = 2.9°
graphite	*h* = −7→7
Non–profiled ω/2θ scans	*k* = −7→7
2155 measured reflections	*l* = −10→10
1078 independent reflections	3 standard reflections every 120 min
755 reflections with *I* > 2σ(*I*)	intensity decay: 2%

### Refinement

**Table:**

Refinement on *F*^2^	Primary atom site location: structure-invariant direct methods
Least-squares matrix: full	Secondary atom site location: difference Fourier map
*R*[*F*^2^ > 2σ(*F*^2^)] = 0.053	Hydrogen site location: inferred from neighbouring sites
*wR*(*F*^2^) = 0.148	H-atom parameters constrained
*S* = 1.03	*w* = 1/[σ^2^(*F*_o_^2^) + (0.0786*P*)^2^ + 0.0033*P*] where *P* = (*F*_o_^2^ + 2*F*_c_^2^)/3
1078 reflections	(Δ/σ)_max_ = 0.001
75 parameters	Δρ_max_ = 0.28 e Å^−^^3^
88 restraints	Δρ_min_ = −0.23 e Å^−^^3^

### Special details

**Table:**

Geometry. All esds (except the esd in the dihedral angle between two l.s. planes) are estimated using the full covariance matrix. The cell esds are taken into account individually in the estimation of esds in distances, angles and torsion angles; correlations between esds in cell parameters are only used when they are defined by crystal symmetry. An approximate (isotropic) treatment of cell esds is used for estimating esds involving l.s. planes.
Refinement. Refinement of *F*^2^ against ALL reflections. The weighted *R*-factor wR and goodness of fit *S* are based on *F*^2^, conventional *R*-factors *R* are based on *F*, with *F* set to zero for negative *F*^2^. The threshold expression of *F*^2^ > σ(*F*^2^) is used only for calculating *R*-factors(gt) etc. and is not relevant to the choice of reflections for refinement. *R*-factors based on *F*^2^ are statistically about twice as large as those based on *F*, and *R*- factors based on ALL data will be even larger.

### Fractional atomic coordinates and isotropic or equivalent isotropic displacement parameters (Å^2^)

**Table:**

	*x*	*y*	*z*	*U*_iso_*/*U*_eq_	Occ. (<1)
B1	−0.001 (3)	−0.298 (2)	0.2892 (18)	0.0268 (10)	0.50
O1	−0.243 (3)	−0.393 (3)	0.318 (3)	0.0399 (19)	0.50
H1	−0.2184	−0.4584	0.4092	0.060*	0.50
O2	0.236 (2)	−0.334 (2)	0.404 (2)	0.0351 (15)	0.50
H2A	0.3669	−0.3158	0.3510	0.053*	0.50
C1	0.0096 (17)	0.113 (2)	−0.1593 (16)	0.0246 (10)	0.50
C2	−0.2061 (18)	−0.068 (2)	−0.1647 (17)	0.0314 (10)	0.50
H2	−0.3512	−0.1021	−0.2661	0.038*	0.50
C3	−0.207 (2)	−0.197 (2)	−0.0217 (17)	0.0314 (10)	0.50
H3	−0.3529	−0.3166	−0.0282	0.038*	0.50
C4	0.0069 (18)	−0.150 (2)	0.1318 (16)	0.0246 (10)	0.50
C5	0.2219 (19)	0.029 (2)	0.1375 (17)	0.0314 (10)	0.50
H5	0.3670	0.0634	0.2389	0.038*	0.50
C6	0.223 (2)	0.158 (2)	−0.0057 (17)	0.0314 (10)	0.50
H6	0.3685	0.2776	0.0010	0.038*	0.50
C7	0.016 (2)	0.256 (2)	−0.3128 (15)	0.0268 (10)	0.50
O3	0.237 (3)	0.341 (3)	−0.344 (3)	0.0399 (19)	0.50
N1	−0.212 (3)	0.283 (3)	−0.415 (3)	0.0351 (15)	0.50
H1A	−0.2112	0.3606	−0.5051	0.042*	0.50
H1B	−0.3631	0.2237	−0.3916	0.042*	0.50

### Atomic displacement parameters (Å^2^)

**Table:**

	*U*^11^	*U*^22^	*U*^33^	*U*^12^	*U*^13^	*U*^23^
B1	0.0340 (13)	0.027 (3)	0.024 (2)	0.0062 (15)	0.0098 (12)	0.011 (2)
O1	0.0295 (7)	0.054 (5)	0.048 (4)	0.002 (2)	0.0081 (18)	0.035 (4)
O2	0.0282 (18)	0.046 (4)	0.0412 (16)	0.006 (2)	0.0081 (15)	0.029 (3)
C1	0.0301 (11)	0.028 (3)	0.020 (3)	0.0076 (11)	0.0089 (11)	0.0097 (19)
C2	0.0331 (11)	0.038 (3)	0.024 (3)	−0.0005 (12)	−0.0004 (12)	0.0137 (19)
C3	0.0329 (11)	0.034 (3)	0.030 (3)	−0.0018 (12)	0.0048 (12)	0.015 (2)
C4	0.0301 (11)	0.028 (3)	0.020 (3)	0.0076 (11)	0.0089 (11)	0.0097 (19)
C5	0.0331 (11)	0.038 (3)	0.024 (3)	−0.0005 (12)	−0.0004 (12)	0.0137 (19)
C6	0.0329 (11)	0.034 (3)	0.030 (3)	−0.0018 (12)	0.0048 (12)	0.015 (2)
C7	0.0340 (13)	0.027 (3)	0.024 (2)	0.0062 (15)	0.0098 (12)	0.011 (2)
O3	0.0295 (7)	0.054 (5)	0.048 (4)	0.002 (2)	0.0081 (18)	0.035 (4)
N1	0.0282 (18)	0.046 (4)	0.0412 (16)	0.006 (2)	0.0081 (15)	0.029 (3)

### Geometric parameters (Å, °)

**Table:**

B1—O1	1.351 (8)	C3—C4	1.391 (8)
B1—O2	1.393 (8)	C3—H3	0.9300
B1—C4	1.546 (6)	C4—C5	1.391 (8)
O1—H1	0.8200	C5—C6	1.384 (8)
O2—H2A	0.8200	C5—H5	0.9300
C1—C6	1.388 (8)	C6—H6	0.9300
C1—C2	1.397 (8)	C7—O3	1.246 (7)
C1—C7	1.505 (6)	C7—N1	1.298 (7)
C2—C3	1.384 (8)	N1—H1A	0.8600
C2—H2	0.9300	N1—H1B	0.8600
			
O1—B1—O2	118.9 (15)	C5—C4—B1	122.2 (8)
O1—B1—C4	119.4 (13)	C6—C5—C4	120.8 (5)
O2—B1—C4	121.6 (12)	C6—C5—H5	119.6
C6—C1—C2	117.8 (5)	C4—C5—H5	119.6
C6—C1—C7	120.0 (7)	C5—C6—C1	121.2 (6)
C2—C1—C7	122.2 (7)	C5—C6—H6	119.4
C3—C2—C1	121.1 (5)	C1—C6—H6	119.4
C3—C2—H2	119.5	O3—C7—N1	120.8 (16)
C1—C2—H2	119.5	O3—C7—C1	120.4 (13)
C2—C3—C4	120.8 (5)	N1—C7—C1	118.8 (13)
C2—C3—H3	119.6	C7—N1—H1A	120.0
C4—C3—H3	119.6	C7—N1—H1B	120.0
C3—C4—C5	118.2 (5)	H1A—N1—H1B	120.0
C3—C4—B1	119.5 (8)		

### Hydrogen-bond geometry (Å, °)

**Table:**

*D*—H···*A*	*D*—H	H···*A*	*D*···*A*	*D*—H···*A*
O1—H1···O2^i^	0.82	1.96	2.77 (2)	167
O2—H2A···O1^ii^	0.82	2.05	2.79 (2)	149
O2—H2A···O3^iii^	0.82	2.00	2.73 (2)	149
N1—H1A···O3^iv^	0.86	2.14	2.97 (3)	160.7
N1—H1B···O1^v^	0.86	2.30	2.97 (2)	135.7
N1—H1B···O3^vi^	0.86	2.18	2.90 (2)	140.8

Symmetry codes: (i) −*x*, −*y*−1, −*z*+1; (ii) *x*+1, *y*, *z*; (iii) −*x*+1, −*y*, −*z*; (iv) −*x*, −*y*+1, −*z*−1; (v) −*x*−1, −*y*, −*z*; (vi) *x*−1, *y*, *z*.
